# The Influence of Noise Floor on the Measurement of Laser Linewidth Using Short-Delay-Length Self-Heterodyne/Homodyne Techniques

**DOI:** 10.3390/mi13081311

**Published:** 2022-08-13

**Authors:** Zhongan Zhao, Zhenxu Bai, Duo Jin, Xiaojing Chen, Yaoyao Qi, Jie Ding, Bingzheng Yan, Yulei Wang, Zhiwei Lu, Richard P. Mildren

**Affiliations:** 1 Center for Advanced Laser Technology, Hebei University of Technology, Tianjin 300401, China; 2 Hebei Key Laboratory of Advanced Laser Technology and Equipment, Tianjin 300401, China; 3 Shanghai Institute for Advanced Communication and Data Science, Shanghai University, Shanghai 200444, China; 4 MQ Photonics Research Centre, Department of Physics and Astronomy, Macquarie University, Sydney, NSW 2109, Australia

**Keywords:** lasers, narrow linewidth, linewidth measurement, noise floor, self-heterodyne

## Abstract

Delayed self-heterodyne/homodyne measurements based on an unbalanced interferometer are the most used methods for measuring the linewidth of narrow-linewidth lasers. They typically require the service of a delay of six times (or greater) than the laser coherence time to guarantee the Lorentzian characteristics of the beat notes. Otherwise, the beat notes are displayed as a coherent envelope. The linewidth cannot be directly determined from the coherence envelope. However, measuring narrow linewidths using traditional methods introduces significant errors due to the 1/*f* frequency noise. Here, a short fiber-based linewidth measurement scheme was proposed, and the influence of the noise floor on the measurement of the laser linewidth using this scheme was studied theoretically and experimentally. The results showed that this solution and calibration process is capable of significantly improving the measurement accuracy of narrow linewidth.

## 1. Introduction

Narrow-linewidth lasers with high spectral purity, high coherence, and low-frequency noise are of great significance for fundamental science research and practical applications [1]. These lasers have been an enabling technology in atomic physics research, nonlinear optics research, high-speed laser communication, lidar, and gravity wave detection [2,3,4,5,6,7]. Narrow-linewidth lasers typically refer to single-frequency lasers, and in these lasers, the linewidth is highly dependent on laser noise characteristics [8,9,10]. Since the noise is difficult to measure directly and various application scenarios have different requirements on laser noise characteristics, the linewidth value is often used to judge the practicability of the laser. The ability to accurately measure laser linewidth has, therefore, become essential to the development and manufacture of narrow-linewidth lasers.

Various techniques have been developed for the measurement of laser linewidth [11]. Although being assisted by another identical laser source is an ideal approach to measuring the laser linewidth [12,13,14], delayed self-heterodyne and self-homodyne are still the most practical methods that have been widely applied for different kinds of lasers at present [15,16,17,18,19,20]. The principle of the self-heterodyne/homodyne method is to generate a beat signal from two high-frequency laser signals from the laser being characterized. This beat signal (with a frequency lower than that of the laser signal) is then measured using a high-resolution electrical spectrum analyzer [21]. In the self-heterodyne/homodyne setup, the optical signals propagating in the two arms of the interferometer must be incoherent to ensure that the beat signal retains the original Lorentzian line shape of the laser [22]. This requires that the delay on one arm of the interferometer is greater than around six times the coherence time of the tested laser [23]. If the delay is insufficient, an envelope will be observed which will affect the measurement accuracy at which the linewidth (−3 dB) can be determined.

In the case of lasers with ultra-narrow linewidths, the coherence time of the laser is typically very high, thus necessitating the use of very long delay fiber lengths. The use of a very long delay ultimately leads to a generation of 1/*f* noise [24], resulting in a spreading of the observed spectrum and significant errors in the linewidth measurement. Several fitting schemes have been developed for use with a long fiber delay [25], but the effect of 1/*f* noise can only be eliminated from the measurements through the use of short delay fibers [26]. With a short delay, the beat note exhibits a characteristic envelope function with multiple extrema (maxima and minima) related to the degree of coherence between the signals in the two interferometer arms. The linewidth can be extracted through analysis of the envelope. Typical linewidth extraction schemes include loop-iterative algorithms based on signal modulation and demodulation [27,28] and equation-solving schemes based on typical values such as amplitude difference of extrema within the envelope [29,30,31,32]. Although these algorithms have significantly improved the resolution and accuracy of techniques employing short-delay-length fibers, experimental data using these algorithms are still subject to the noise floor measurement, which can limit the accuracy of the linewidth measurement technique.

In this paper, we proposed a narrow linewidth measurement scheme using short-delay self-heterodyne interferometry and analyzed the impact of the noise floor on this scheme both theoretically and experimentally. The results showed that, compared with the conventional scheme, this short fiber-based approach can significantly enhance the linewidth measurement accuracy after the optimization of the noise floor. The linewidth algorithm and the noise floor avoidance in this paper provide a beneficial technique for further improving the accuracy of linewidth measurement.

## 2. Materials and Methods

**Figure 1:**
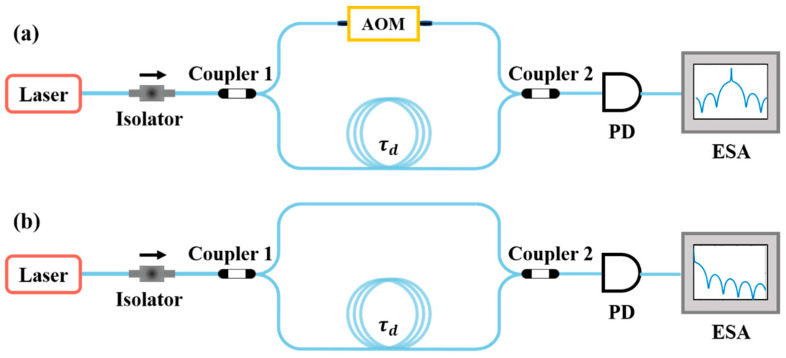
Schematics showing the structures of unbalanced interferometers employed for laser linewidth measurement using a delayed self-heterodyne technique (**a**) and a delayed self-homodyne technique (**b**).

Delayed self-heterodyne and delayed self-homodyne methods based on an unbalanced interferometer are shown schematically in Figure 1. The difference between the two structures is that in the delayed self-heterodyne setup, an acousto-optic modulator is included in the non-delay arm to implement frequency shifting. The delayed self-heterodyne process can be expressed by a mathematical model. In this case, the beat signal which reaches the electrical spectrum analyzer (ESA) can be expressed as [22,30,31]:

(1) $$S=S_{1}\times S_{2}+S_{3}$$

(2) $$S_{1}=\frac{P_{0}{}^{2}}{4\pi }\frac{\Delta f}{\Delta f^{2}+{\left(f-f_{1}\right)}^{2}}$$

(3) $$S_{2}=1-exp\left(-2\pi \tau_{d}\Delta f\right)\left[\cos\left(2\pi \tau_{d}\left(f-f_{1}\right)\right)+\Delta f\frac{sin\left(2\pi \tau_{d}\left(f-f_{1}\right)\right)}{f-f_{1}}\right]$$

(4) $$S_{3}=\frac{\pi P_{0}{}^{2}}{2}exp\left(-2\pi \tau_{d}\Delta f\right)\delta \left(f-f_{1}\right)$$

where $P_{0}$ is the mixed optical power, Δf is the laser linewidth, $f_{1}$ is the modulation frequency of the AOM, $\tau_{d}$ is the fiber delay time, and δ(f) is the impulse function. The relationship between the linewidth and the coherence time $\tau_{c}$ of a laser is given by:

(5) $$\Delta f=\frac{1}{\pi \;\tau_{c}}$$

**Figure 2:**
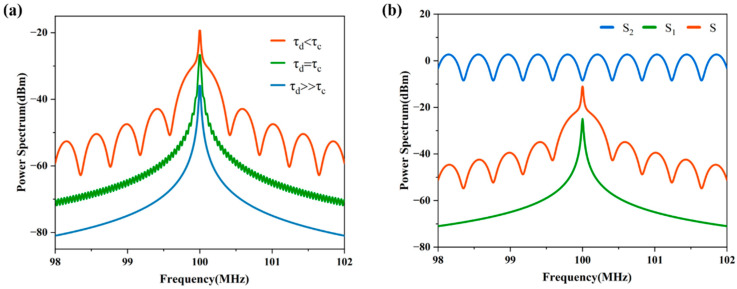
Theoretical plots of the PSD from a delayed self−heterodyne measurement setup showing the effects of different delay times (**a**) and the line shapes of $S_{1}$, $S_{2},$ and S (**b**).

The theoretically calculated power spectral densities (PSDs) of the signal output (as would be recorded on the ESA) for different fiber lengths are shown in Figure 2a. The shorter the fiber length, the more prominent the envelope function becomes; the longer the fiber, the more obvious the Lorentzian characteristic of the spectrum. Three terms make up the PSD, that is, $S_{1}$ is the Lorentz function, $S_{2}$ is a periodic function, and $S_{3}$ is the impulse function. Since $S_{3}$ only impacts the center frequency of the beat signal (the frequency in the delayed self-heterodyne being $f_{1}$ and in the delay self-homodyne being 0), with the value being zero at all other frequencies, the effect of $S_{3}$ on the PSD can be ignored in the whole frequency domain. The spectral line of S is equivalent to the modulated signal generated after $S_{2}$ modulates $S_{1}$, with $S_{1}$ as the modulated signal and $S_{2}$ as the carrier signal; this effect is plotted in Figure 2b. The changes in fiber length only affect the $S_{2}$ term in the PSD function, and its period is only controlled by the fiber length. When the fiber is short, the amplitude and the period are more prominent and the modulation effect is more obvious, with this being apparent in the envelope of the PSD. The analysis of these PSD functions with highly modulated envelopes has been the focus of significant research and has led to the development of several algorithms for extracting the laser linewidth. These algorithms are, however, still subject to the noise properties of the measurement system and in particular the noise floor of the beat signal measurement using the ESA.

Current methods which are used to extract the laser linewidth from the PSD envelope mainly revolve around the application of demodulation within calculations. In this work, we applied a scheme whereby different combinations of extrema (maxima and minima) in the signal PSD are analyzed for the determination of the laser linewidth. We developed a function that takes into consideration the amplitude difference of extrema within the PSD and the laser linewidth:

(6) $$\begin{array}{ll} \Delta s=10log_{10}S_{H}-10log_{10}S_{L} & \\ =10log_{10}\frac{\left[\Delta f^{2}+{\left[\frac{\left(k+2\right)c}{2nL}\right]}^{2}\right]}{\left[\Delta f^{2}+{\left[\frac{\left(m+2\right)c}{2nL}\right]}^{2}\right]}\frac{\left[1-exp\left(-2\pi \frac{nL}{c}\Delta f\right)\cos\left[\left(m+2\right)\pi \right]\right]}{\left[1-exp\left(-2\pi \frac{nL}{c}\Delta f\right)\cos\left[\left(k+2\right)\pi \right]\right]} & \end{array}$$

where Δs is selected as the magnitude difference between the two extreme points, $S_{H}$ is the larger value of the two extreme points, $S_{L}$ is the smaller value of the two points, that is, $S_{H}>S_{L}$, and m and k are natural numbers, with m=0 representing the nearest extreme point from the center frequency (the first minima of the envelope) and m=1 representing the second nearest extreme point from the center frequency (the first maxima adjacent to the center frequency), noting that m≠k, and as two extrema are adjacent, |k−m=1|.

**Figure 3:**
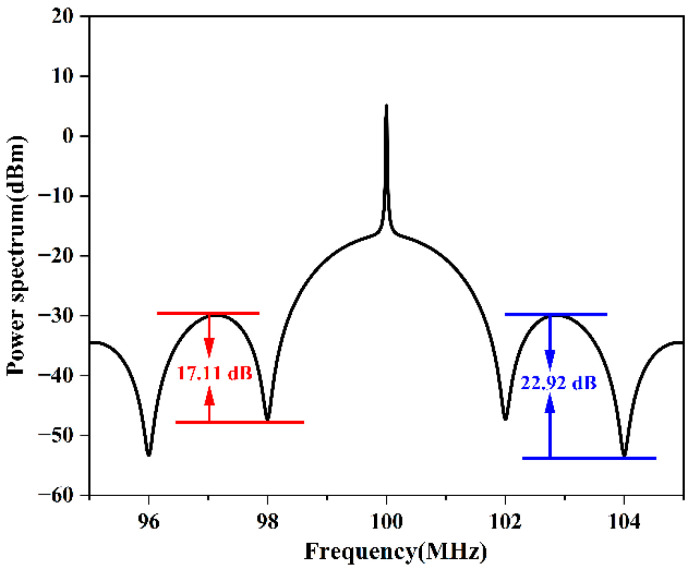
Plot of the simulated PSD function using a 100 m long fiber delay line.

We first undertook a theoretical investigation of the analysis technique and used the parameters of linewidth = 5.5 kHz and fiber length = 100 m to produce a simulated PSD; the result is shown in Figure 3. We then used the solving function (Equation (6)) and the simulated PSD to extract the linewidth. The results using two different pairs of extrema are summarized in Table 1.

## 3. Experimental Results and Analysis

**Figure 4:**
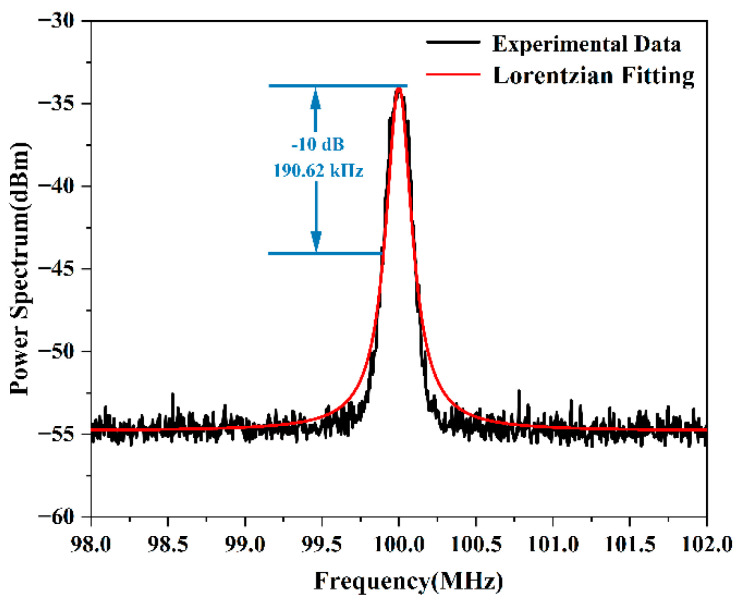
Plot of the experimentally obtained PSD using a 50 km long delay fiber.

Our experimental measurements of laser linewidth were conducted using a commercial narrow-linewidth laser (RIO0175-5-07-1, which had a manufacturer-specified integral linewidth of 5.1 kHz). We first performed measurements on this commercial laser using a conventional scheme. The experimentally measured PSD is shown in Figure 4. The −10 dB bandwidth was taken, and the laser linewidth was calculated to be 31.77 kHz by the relationship of power loss. This obtained laser linewidth was much larger than the integrated linewidth given in the manufacturer’s report. The conventional scheme does introduce excessive 1/*f* frequency noise.

**Figure 5:**
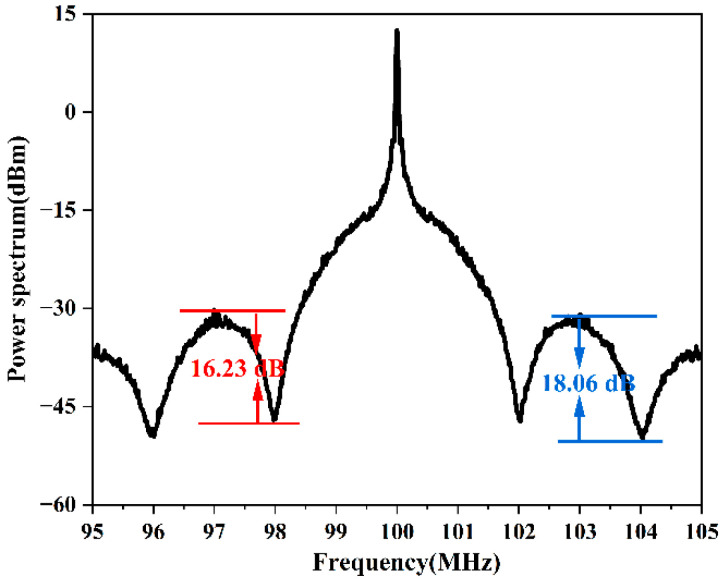
Plot of the experimentally obtained PSD using a 100 m long delay fiber.

We then replaced the long fiber with a 100 m short fiber and measured the laser linewidth using our method as the theoretical calculation (Equation (6)). The linewidth of the laser used in the experiment was calculated using two pairs of extrema. The experimentally measured PSD is shown in Figure 5, and the results are summarized in Table 2.

Linewidths on the order of kilohertz or narrower are difficult to measure directly; therefore, the manufacturing report usually provides a noise integration linewidth. Our scheme can directly measure narrow linewidths on the order of kilohertz. Considering the inherent error between different schemes, the obtained linewidth of 6.61 kHz using our scheme is reasonable; however, the result of 17.34 kHz apparently showed an error. Therefore, analyzing the reasons for the difference between 17.34 kHz and 6.61 kHz is critical to further improving the accuracy of our scheme. In contrast, the accuracy of our scheme was significantly improved, but there are still some slight errors. According to the results shown in Figure 3 and Table 1, taking the amplitude differences at different sets of extrema on the PSD envelope should yield the same result. However, in the experimental results shown in Figure 5 and Table 2, different linewidths were calculated when using different sets of extrema. Based on these results, it is apparent that the closer to the center frequency the extrema, the smaller the calculated amplitude difference and the smaller the laser linewidth (the linewidth being closer to the actual linewidth).

**Figure 6:**
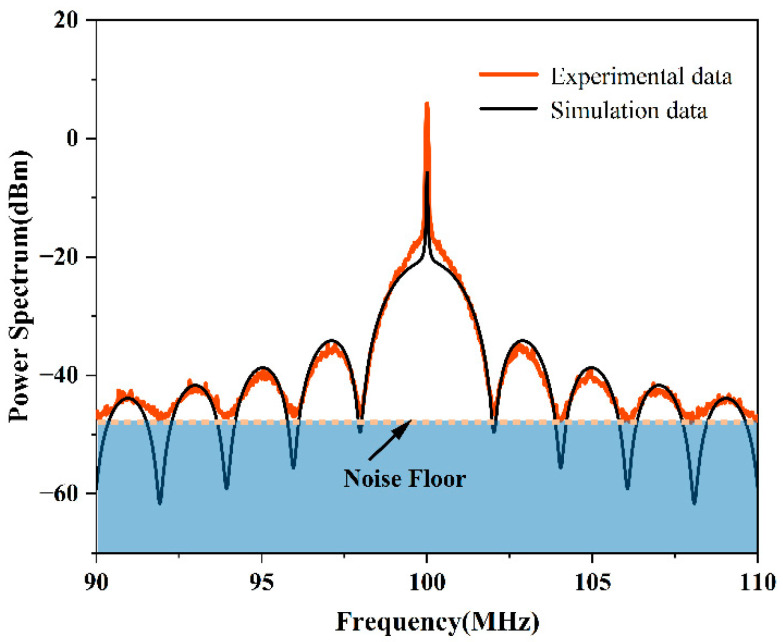
Plots showing the impact of the noise floor measurement and the resultant differences between the simulated and experimental data.

In the experimentally obtained PSD, the amplitude difference far from the center frequency was smaller than the ideal, and the calculated linewidth result was larger than the actual value. We believe the noise floor of the measurement was responsible for this difference. The noise floor in essence defined the minimum signal strength that can be perceived by the receiver and the ESA. When the signal strength input to the receiver was less than this threshold, the signal was submerged. Because the beat signal intensity was distributed over a wide frequency range and the maxima and minima occurred at progressively lower intensities, parts of the measured data became buried in the noise floor. As a result of the noise floor, the experimentally measured values of the minima of the function were not reflective of the real value. The values of the minima were in fact lower in amplitude than their true values, and as a result, the calculated amplitude difference was smaller and the calculated linewidth was larger than the true value. This effect is shown schematically in Figure 6.

Figure 6 overlays the simulated and experimental results for a device with the same parameters and shows the way that the noise floor impacted the experimental measurements. The plot showed that the further away from the center frequency of the PSD the extrema, the more serious the submergence of the minima of the envelope and the larger the difference between the experimental and theoretical values.

## 4. Avoidance of Noise Floor Effects

The noise floor is an inherent property of the measurement system and cannot be eliminated, but its influence on the measurement can be minimized through judicious choice of experiment parameters and setups, to ensure that the maximum number of extrema (especially the minima) are above the noise floor. There are several strategies that can be used to achieve this.

### 4.1. Using a Delayed Self-Heterodyne Setup

**Figure 7:**
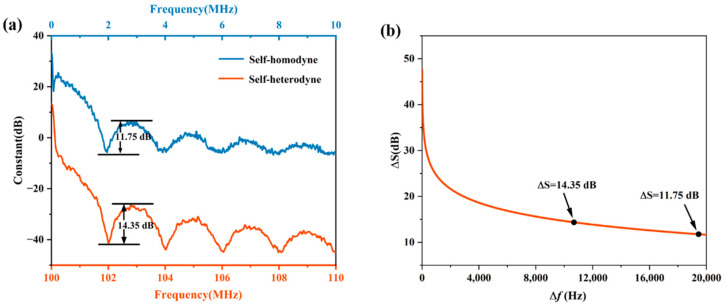
Plots of experimental data comparing signal measurement results taken from a commercial laser (RIO0175-5-07-1) obtained using the self-heterodyne and self-homodyne setups. (**a**) Plots of the signal PSD. (**b**) Plots of the difference in extrema amplitude (difference between the maxima and minima values) as a function of the theoretical linewidth of the laser for the extrema closest to the center frequency.

Delayed self-heterodyne and delayed self-homodyne setups are both based on unbalanced interferometers. Their theoretical models are identical, except for the modulation induced by the AOM. The linewidth calculation algorithm using short-delay-length fibers is also applicable to both structures. Under the same conditions, the self-heterodyne method, in comparison to the self-homodyne method, produces a signal spectrum much closer to that which is expected by theory. This can be seen in the plots of Figure 7a, where the signal spectra taken using the self-heterodyne and self-homodyne techniques are plotted for the same test laser (RIO0175-5-07-1, Redfern Integrated Optics Inc., Santa Clara, CA, USA). The signal spectrum recorded using the self-heterodyne setup was of higher fidelity, clearly showing the distinct spike at the center frequency (left most of the plot) and more distinct extrema (maxima and minima). According to the relationship between the amplitude difference and the linewidth plotted in Figure 7b, the linewidth result calculated using the self-heterodyne setup was smaller and closer to the true linewidth of the laser in comparison to that calculated using the self-homodyne setup.

### 4.2. Making Use of the Extrema Closest to the Center Frequency

As depicted in Figure 6, the reliability of experimental data further from the center frequency of the signal plot decreased because of the noise floor. Hence, it is apparent that using data as close to the center frequency as possible yielded the most reliable results. It has also been verified in the results of Table 2.

### 4.3. Increasing the Injected Laser Power or Using an Amplifier

**Figure 8:**
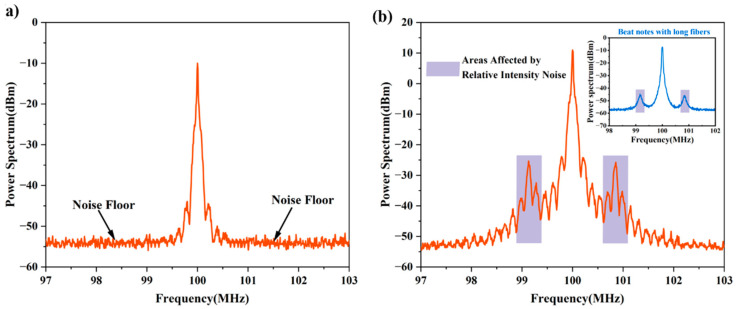
Plots of the self-heterodyne signal PSD taken for a power-scalable fiber laser (CONNET, CoSF-D-YB-M) for output powers of 1 mW (**a**) and 10 mW (**b**) (the Lorentzian-shaped spectrum as recorded using a long length of delay fiber (50 km) is shown as the inset).

The noise floor limits the minimum signal that the system can handle. It is, thus, logical to increase the signal power as a means of ensuring that the signal received by the detection system is above the noise floor. Depending on the device under test, this may be achieved simply by increasing its output power or through the application of amplifiers such as a fiber amplifier [33]. A caveat is that the addition of fiber amplifiers often introduces frequency noise with 1/*f* characteristics [34]. In this work, we have investigated the effect that increasing the output power of a laser under test has on the detected self-heterodyne signal. Here, we have tested a power-tunable narrow-linewidth fiber laser (CoSF-D-YB-M, Connet Laser Technology Co., Ltd., Shanghai, China) with a 1000 m long delay fiber. Plots presenting the self-heterodyne signal PSDs for two power output levels of 1 mW and 10 mW are shown in Figure 8a,b, respectively.

When the output power of the fiber laser was set to 1 mW, a majority of the recorded signal spectrum was submerged under the noise floor (except the first-order maxima). In comparison, when the output power was set to 10 mW, a significant number of maxima and minima became discernable in the recorded signal spectrum with fewer extrema submerged in the noise floor. The amplitude difference between the first valley and the second peak did not change significantly; therefore, the linewidth did not change remarkably after power fine-tuning (according to Equation (6)). However, the difference between the second peak and the second valley increased from 9.20 dB to 15.35 dB. It means that the second-order envelope at low power could not be completely displayed due to the influence of the noise floor. The measurement result after the power increased to 10 mW was 3.54 kHz, which corresponded to the linewidth range of 1–10 kHz provided in the manufacturing report. With more data above the noise floor, the number of envelopes increased and the envelope became clearer at the same time. Therefore, it is suitable for various linewidth extraction schemes based on coherent envelopes [27,28,29,30,31]. It should be noted that two secondary maxima were observed on either side of the center frequency of the signal spectrum (these are highlighted in the plot of Figure 8b). These are a result of intensity noise, and these features should be avoided when deciding on which extrema should be used for linewidth calculation.

It should be noted that while increasing laser power can be used to ensure the detected signal is above the noise floor of the measurement system, care must be taken to ensure that the laser power is below the damage threshold of components within the setup.

### 4.4. Tailoring the Length of Delay Fiber

**Figure 9:**
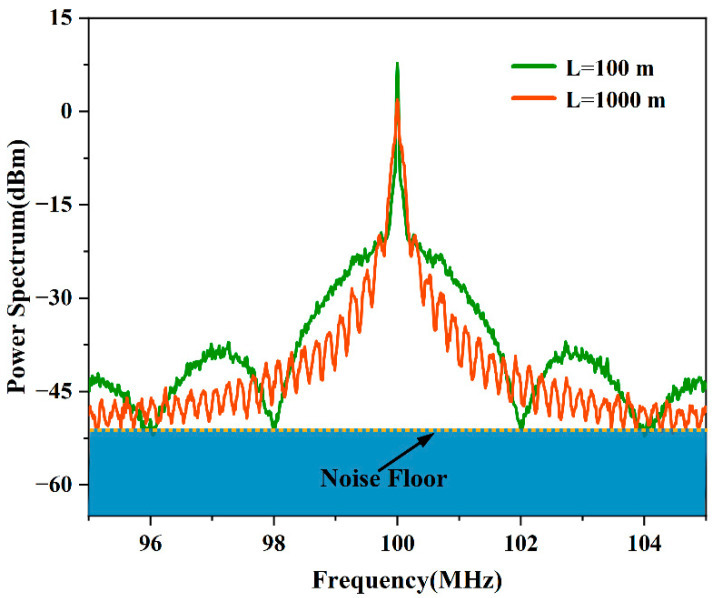
Plots showing the experimentally obtained signal PSD functions for delay fiber lengths of 100 m and 1000 m.

In the instance where the input laser power cannot be increased, there is still an opportunity to improve the quality of the detected signal by changing the length of the delay fiber. Increasing the delay fiber length reduces the period and the amplitude of S_2_, resulting in the development of more extrema for a given frequency span. The Lorentzian characteristic of the PSD also becomes more apparent, and the amplitude of the extrema increases. Figure 9 shows the PSD recorded for the narrow-linewidth semiconductor laser (RIO0175-5-07-1, Redfern Integrated Optics Inc., Santa Clara, CA, USA) using two delay fiber lengths of 100 m and 1000 m and using the same sweep span (10 MHz). The PSD recorded using the 1000 m length of delay fiber had significantly more extrema above the noise floor of the measurement system. The recorded spectra were analyzed, and a linewidth of 16.04 kHz was determined using the 100 m long delay fiber, with an amplitude difference of 12.59 dB between the extrema closest to the center frequency. A linewidth of 6.40 kHz was determined using the 1000 m long delay fiber with an amplitude difference of 12.63 dB for the second set of extrema from the center frequency. We effectively avoided the influence of the noise floor by tailoring the fiber length, and the measurement result at the 1000 m delay (6.40 kHz) was more accurate than that with the 100 m delay (6.61 kHz).

In the above experiments, using the amplitude difference taken between the second set of extrema from the center frequency was more accurate than using the 1000 m long delay fiber. This was because as the fiber length increased, the period of the signal envelope became smaller and the first maxima occurred progressively closer to the center frequency and the impulse function of the response. Therefore, care must be taken in the analysis of the recorded signal spectrum and the most appropriate set of extrema chosen on an almost case-by-case basis. In the case where a delay fiber length of 100 m was used, the extrema closest to the center frequency was used, as these points were unaffected by the Lorentzian response of the signal and were the extrema most above the noise floor. In the case where a 1000 m long delay fiber was used, when the extrema closest to the center frequency was well above the noise floor, they were affected by the Lorentzian response of the signal.

## 5. Conclusions

In conclusion, we have investigated the application of short delay fiber lengths in delayed self-heterodyne laser linewidth measurements using theoretical and experimental approaches. We found that while the application of short lengths of delay fiber can be very effective in avoiding 1/*f* noise, which is known to impact measurements using long lengths of delay fiber, care must however be taken when analyzing the PSD of the signal recorded using short lengths of delay fiber, especially for the effect of the noise floor measurement. Based on our results, we have taken a step-wise approach to highlight the impact of the noise floor and have offered a range of scenarios by which its effects on the final linewidth calculation can be minimized. By studying the noise floor, the measurement accuracy of the short-fiber delayed self-heterodyne method has been successfully improved compared to the initial results. After a series of experimental comparisons, we successfully improved the accuracy of the algorithm in actual measurements. The measured linewidth of the RIO laser was optimized from the initial frequency of 16.04 kHz to 6.40 kHz, which was closer to the real level of the laser linewidth. Ultimately, the application of a short length of delay fiber in a delayed self-heterodyne laser linewidth measurement was used very effectively as a means of avoiding 1/*f* noise, provided that care was taken in the analysis of the recorded signal PSD and with mind to conditions under which the PSD of the signal was recorded. We believe that the methodology and results presented in this work represent a significant step towards the development of more reliable, compact, and effective methods for the measurement of the linewidth of narrow-linewidth lasers.

## Figures and Tables

**Table 1:** Summary of results obtained through the analysis of the theoretical PSD plotted in Figure 3, using two different sets of extrema.

*m*	*k*	ΔS (dB)	Δf (kHz)
1	0	17.11	5.504
1	2	22.92	5.502

**Table 2:** Summary of results obtained through analysis of the experimentally obtained PSD using two different sets of extrema.

*m*	*k*	ΔS (dB)	Δf (kHz)
1	0	16.23	6.610
1	2	18.06	17.340

## Data Availability

No new data were created.
